# Territorially Stratified Modeling for Sustainable Management of Free-Roaming Cat Populations in Spain: A National Approach to Urban and Rural Environmental Planning

**DOI:** 10.3390/ani15152278

**Published:** 2025-08-04

**Authors:** Octavio P. Luzardo, Ruth Manzanares-Fernández, José Ramón Becerra-Carollo, María del Mar Travieso-Aja

**Affiliations:** 1 Research Institute of Biomedical and Health Sciences (IUIBS), University of Las Palmas de Gran Canaria, Paseo Blas Cabrera “Físico” s/n, 35016 Las Palmas de Gran Canaria, Spain; marimar.travieso@ulpgc.es; 2 Spanish Biomedical Research Center in Physiopathology of Obesity and Nutrition (CIBERObn), Avenida Monforte de Lemos, 5, 28029 Madrid, Spain; 3 General Directorate for Animal Rights, Ministry of Social Rights and the 2030 Agenda, Government of Spain, Paseo del Prado 18, 28014 Madrid, Spain; rmanzanares@dsca.gob.es (R.M.-F.); jbecerra@dsca.gob.es (J.R.B.-C.)

**Keywords:** trap–neuter–return (TNR), free-roaming cats, animal welfare policy, population modeling, population management, One Health

## Abstract

This study presents the scientific and methodological foundation of Spain’s first national framework for the ethical management of community cat populations: the Action Plan for the Management of Community Cat Colonies (PACF), launched in 2025 under the mandate of Law 7/2023. This pioneering legislation introduces a standardized, nationwide obligation for trap–neuter–return (TNR)-based management of free-roaming cats, defined as animals living freely, territorially attached, and with limited socialization toward humans. The PACF aims to support municipalities in implementing this mandate through evidence-based strategies that integrate animal welfare, biodiversity protection, and public health objectives. Using standardized data submitted by 1128 municipalities (13.9% of Spain’s total), we estimated a baseline population of 1.81 million community cats distributed across 125,000 colonies. These data were stratified by municipal population size and applied to national census figures to generate a model-ready demographic structure. We then implemented a stochastic simulation using Vortex software to project long-term population dynamics over a 25-year horizon. The model integrated eight demographic–environmental scenarios defined by a combination of urban–rural classification and ecological reproductive potential based on photoperiod and winter temperature. Parameters included reproductive output, mortality, sterilization coverage, abandonment and adoption rates, stochastic catastrophic events, and territorial carrying capacity. Under current sterilization rates (~20%), our projections indicate that Spain’s community cat population could surpass 5 million individuals by 2050, saturating ecological and social thresholds within a decade. In contrast, a differentiated sterilization strategy aligned with territorial reproductive intensity (50% in most areas, 60–70% in high-pressure zones) achieves population stabilization by 2030 at approximately 1.5 million cats, followed by a gradual long-term decline. This scenario prioritizes feasibility while substantially reducing reproductive output, particularly in rural and high-intensity contexts. The PACF combines stratified demographic modeling with spatial sensitivity, offering a flexible framework adaptable to local conditions. It incorporates One Health principles and introduces tools for adaptive management, including digital monitoring platforms and standardized welfare protocols. While ecological impacts were not directly assessed, the proposed demographic stabilization is designed to mitigate population-driven risks to biodiversity and public health without relying on lethal control. By integrating legal mandates, stratified modeling, and realistic intervention goals, this study outlines a replicable and scalable framework for coordinated action across administrative levels. It exemplifies how national policy can be operationalized through data-driven, territorially sensitive planning tools. The findings support the strategic deployment of TNR-based programs across diverse municipal contexts, providing a model for other countries seeking to align animal welfare policy with ecological planning under a multi-level governance perspective.

## Simple Summary

Free-roaming cats are present in urban and rural areas across Spain, often forming colonies that raise concerns about animal welfare, biodiversity, and public health. In response to these challenges, Spain adopted Law 7/2023, which mandates humane management of community cats through trap–neuter–return (TNR). This study presents the scientific basis of the national strategy developed to implement this law: the Action Plan for the Management of Community Cat Colonies (PACF). Using data from over 1100 municipalities and a 25-year population model, we show that current sterilization efforts are insufficient to control cat population growth. However, a territorially adapted TNR strategy—tailored to local reproductive conditions—can stabilize and reduce the cat population without resorting to lethal methods. The plan offers a replicable model that combines animal welfare, ecological responsibility, and public health goals.

## 1. Introduction

Urban and peri-urban environments globally face growing challenges in managing free-roaming domestic cats—referred to as community cats under Spain’s legislation [1,2]. These animals intersect diverse concerns: biodiversity conservation, public health, urban sanitation, and animal welfare. Studies document their impact on native species via predation and behavioral disruption, especially in fragmented ecosystems and insular settings [3,4,5,6]. Hybridization with wildcats is another potential concern, although studies suggest that in the Iberian Peninsula, low hybridization rates are observed due to strong spatial segregation between domestic and wildcats [7]. However, in urban contexts, their ecological role is more nuanced, as many rely heavily on anthropogenic food sources. Urban parks and gardens can serve as important refuges for native wildlife but may also become population sinks when exposed to sustained predation pressure from free-roaming cats [8,9,10,11].

Urban and rural environments influence the diet, ecological impact, population densities, reproductive behavior, and social structure of community cats. Urban colonies, often more stable and denser, exhibit prolonged reproductive activity due to year-round food availability, which contributes to social conflicts. In contrast, rural cats show more seasonal reproduction, lower densities, and a greater reliance on hunting [12,13,14,15].

High densities of community cats exacerbate public nuisances, zoonotic and feline disease transmission, and resident conflicts [16,17,18], fueled by abandonment, unregulated feeding practices, and weak institutional oversight. Consequently, there is growing interest in socially acceptable, ethically grounded strategies for urban cat management [19,20]. This trend aligns with international calls for sustained, data-driven, and ethically consistent TNR approaches that integrate monitoring, community engagement, and long-term funding strategies [21].

Among non-lethal control methods, trap–neuter–return (TNR) has become the most widely implemented and socially accepted strategy, particularly in Europe and parts of North America, although it remains controversial in some jurisdictions [22]. It involves humane trapping, sterilization, deworming, vaccination, and return of cats to their territories. When conducted intensively and consistently, TNR can stabilize or reduce populations over time [23,24,25,26]. Case studies from Rome, Tel Aviv, Chicago, and Zurich support its effectiveness and emphasize the role of institutional support and community involvement [24,27,28,29]. Similar successes have been reported in Spanish urban programs [30], reinforcing TNR’s relevance across sociopolitical contexts.

However, TNR’s long-term success is jeopardized by insufficient sterilization rates, irregular follow-up, unmanaged immigration, and poor inter-institutional coordination [21,24,31]. Addressing these limitations requires not only technical precision but also sustained investment, structured governance, and integration with broader urban planning. Boone and LeBaron [21] emphasize that fragmented or short-term efforts often fail to achieve durable outcomes, and highlight the importance of robust metrics, clear policy mandates, and cross-sectoral collaboration.

Spain’s approach to managing community cats has historically been fragmented. Between 2002 and 2020, various autonomous communities and municipalities recognized feline colonies and promoted TNR [32,33,34,35,36]. Yet, most initiatives remained small scale, underfunded, and heavily reliant on volunteers. This landscape shifted with the enactment of Spain’s Animal Welfare Law 7/2023, which introduced a mandatory, nationwide framework for the identification, registration, and TNR-based management of community cats [1,37]. The law defines these animals as individuals of *Felis catus* living freely, territorially attached, and exhibiting low or absent sociability toward humans, while remaining partially dependent on human-provided resources. Municipalities are now required to implement colony registration, census-taking, sterilization, vaccination, deworming, and health monitoring, while lethal control and confinement are explicitly prohibited except under exceptional conditions. This positions Spain as one of the few countries with a national legal obligation for TNR-based management. Comparable legislation exists in Italy’s Framework Law 281/1991, which recognized and protected free-roaming cats and promoted sterilization, although without establishing a comprehensive national system [38]. The Italian law is cited not for its recentness but because it represents a rare legal precedent in Europe: it explicitly prohibits the killing of community cats and mandates non-lethal management. However, its implementation has remained largely decentralized, with municipalities and local animal welfare groups playing a predominant role. In contrast, Spain’s 2023 law establishes a standardized, enforceable framework at the national level, making it a significant step forward in terms of both legal clarity and administrative responsibility.

To operationalize this legal mandate, the Ministry of Social Rights and 2030 Agenda has developed the Action Plan for the Management of Community Cat Colonies (*Plan de Acción sobre Gestión de Colonias Felinas y Gatos Comunitarios*, PACF) in 2025. This technical and management framework provides scientifically grounded guidance for municipalities, integrating ecological, demographic, logistical, and economic considerations, adaptable to different municipal contexts. The population modeling for the Plan is grounded in data submitted by approximately 1.100 municipalities. (13.9% of Spain’s total), which contributed to estimating national community cat populations, modeling demographic trajectories across different management scenarios, and projecting associated costs. These projections are based on the best available evidence from the scientific literature, field data, and case studies both within and beyond Spain. Effective TNR planning requires the integration of baseline diagnostics, demographic modeling, and cost projections to guide strategic investments and ensure long-term monitoring of outcomes [21].

This article presents the scientific rationale and methodological foundations of the PACF. It offers a comprehensive analysis of demographic modeling strategies and strategic recommendations designed to support evidence-based decision-making for community cat management. By situating Spain’s experience within the global context of TNR implementation, this study contributes to broader academic and policy discussions on integrating animal welfare and biodiversity conservation within urban and landscape planning. It underscores the need for management strategies that are ecologically sound, legally robust, and socially legitimate.

## 2. Materials and Methods

### 2.1. Data Sources

The demographic baseline for the PACF was derived from standardized administrative data submitted by 1128 municipalities through the 2024 national funding call issued by the Spanish Directorate-General for Animal Rights [39]. This process, aimed at supporting the development of local cat colony management programs, required municipalities to submit verified census data on community cats and colonies.

Although the 1128 responding municipalities represent only 13.9% of Spain’s total (8131), they include a broad range of sizes and territorial contexts, encompassing more than 50% of the national human population. Given Spain’s administrative structure—divided into 17 autonomous communities, 50 provinces, and over 8000 municipalities—this sample size is sufficient for robust national projections, even if small, sparsely populated municipalities are slightly underrepresented.

Municipalities were stratified into 13 demographic categories based on population size, ranging from fewer than 100 to more than 50,000 inhabitants. Within each category, two key ratios were calculated: the average number of community cats per inhabitant and the average number of cats per colony. These ratios were applied to official population figures from the Spanish National Institute of Statistics to extrapolate national estimates of cat populations and colonies.

The data, processed using standardized Excel spreadsheets, served as the foundation for demographic modeling and economic projections. Although smaller municipalities were underrepresented, stratified modeling mitigated this bias, generating scalable and policy-relevant estimates of Spain’s free-roaming cat population. These estimates provided the demographic structure for long-term projections of population dynamics, which were modeled over a 25-year horizon to reflect the expected duration of national implementation and its ecological inertia.

### 2.2. Population Modeling and Scenario Simulations

To project the evolution of Spain’s community cat population under different intervention strategies, we implemented a stochastic simulation using Vortex [40], a widely used platform for wildlife population viability analysis. While originally designed for species with longer reproductive cycles, Vortex’s capacity to model stochastic events, demographic parameters, and subpopulation dynamics makes it appropriate for comparative scenario analysis in this context. The model simulated population dynamics over a 25-year horizon. Key parameters included reproductive capacity, mortality, sterilization, and stochastic events, with 1000 probabilistic iterations per scenario.

A crucial methodological step was the territorial and ecological stratification of Spanish municipalities. This stratification combined two dimensions: population size and climatic suitability for winter estrus. Based on municipal census data from the Spanish National Statistics Institute [41,42], (INE, 2021) municipalities were classified as urban (U) (more than 20,000 inhabitants) or rural (R) (20,000 inhabitants or fewer), reflecting observed differences in colony structure, density, and reproductive behavior.

Simultaneously, provinces were categorized into four reproductive intensity levels—low (L), medium (M), high (H), and very high (VH)—based on mean daylight hours in December and average winter temperatures [43,44,45,46]. This produced eight demographic–environmental scenarios: UL, UM, UH, and UVH for urban areas, and RL, RM, RH, and RVH for rural ones. Each municipality was assigned to one of these scenarios accordingly. The territorial classification map used to inform model parameters is presented in Supplementary Figure S1.

For each of these eight scenarios, tailored reproductive parameters were defined, including the number of litters per year, the expected size of each litter, and the kitten survival rate. These parameters were derived using a two-stage adjustment modulation process, incorporating both biological potential and environmental correction factors. The theoretical maximum of three litters per female per year was adjusted downward using the concept of the Reproductive Utilization Rate (RUR), reflecting the proportion of fertile periods that result in actual pregnancies. RUR values were set at 0.55 for RL, 0.65 for RM and UL, 0.75 for RH and UM, and 0.85 for RVH, UVH and UH scenarios, according to observed stressors, predation, access to mates, and overall ecological stability.

The effective success probability for each scenario (p_modulated) was calculated by multiplying the base biological success probability per fertile attempt (*p* = 0.85) [47] by the Reproductive Utilization Rate (RUR), which adjusts for environmental and social conditions affecting reproductive success. This yields the formula:

p_modulated = 0.85 × RUR.

To determine the likelihood of a female producing a given number of litters per year, a binomial distribution was used, assuming three possible fertile opportunities per year (*n* = 3). The probability (*P*) of having *k* successful litters was calculated using the following formula:

P litters= nk·pk·1−pn−k

where (*n*, *k*) represents the number of possible combinations, *p* is the effective (modulated) success probability per attempt, and *k* ranges from 0 to 3 litters per year. The resulting distribution allows for the estimation of realistic scenario-specific probabilities for 0, 1, 2, or 3 litters per reproductive female annually. These probabilities are presented in the Results section.

In parallel, the number of kittens per litter was modeled separately for urban and rural municipalities. A normal (Gaussian) distribution was used to estimate the probability of a female producing between 1 and 6 kittens per litter. The distribution was defined by the following equation:

$$f\left(k\right)=\frac{1}{\sigma \sqrt{2\pi }}e^{-\frac{{\left(k-\mu \right)}^{2}}{{2\sigma }^{2}}}$$

where *k* is the number of kittens per litter, *μ* is the expected mean number of kittens (3.75 in urban environments and 4.75 in rural ones [43,47]), and *σ* is the standard deviation (1.2 and 1.3, respectively), accounting for natural variability around the mean.

The calculated values for each *k* (from 1 to 6) were normalized by dividing by the sum of all values (*T*) and then multiplying by 100 to obtain relative probabilities:

$$P\left(k\right)normalized\;=\frac{f(k)}{T}\times 100$$

These scenario-specific probabilities were then used to model the number of viable kittens per female per year, depending on the municipality type.

Kittens were then subjected to age-specific mortality filters to determine the number of viable offspring reaching adulthood. We applied standardized rates: 65% mortality for kittens (<1 year) and 15% for adults [48,49], uniformly across all scenarios to ensure comparability. The output of this multi-step process—litters per female, litter size, and survival probability—allowed the derivation of expected viable offspring per female per year, stratified by province and municipality type.

Each simulation scenario was initialized using population estimates derived from the municipal dataset described in Section 2.1. These baseline populations were configured with 20% of individuals already sterilized, which corresponds to the sterilization assumption consistent with the Plan’s trend-based scenario. Although this figure provides a useful reference point, it does not reflect uniform sterilization coverage across regions. Therefore, it was used as a starting condition upon which additional sterilization efforts were simulated, gradually increasing coverage to test a range of intervention scenarios between 20% and 100%. These successive increments formed the basis of the results presented and discussed in the following sections.

To simulate the effect of annual sterilization campaigns, the “dispersal” function in Vortex was adapted to model the annual transfer of individuals from the unsterilized to the sterilized subpopulation. This approach allowed dynamic modeling of reproductive suppression, without removing individuals from the population.

In addition, the model incorporated stochastic catastrophic events such as panleukopenia outbreaks, poisoning, or mass mortality due to human action, occurring with a 5% annual probability. When triggered, these events applied a 30% increase in mortality across all age classes.

To account for anthropogenic dynamics, two additional processes were introduced. First, an annual influx of abandoned cats was modeled based on survey data from approximately 500 Spanish municipalities collected by the Spanish Directorate of Animal Rights in 2024. These data yielded a national estimate of 109,000 cat abandonments per year, distributed proportionally across the eight environmental–demographic scenarios. Second, the annual adoption of community cats was modeled using the “harvest” function in Vortex, which permanently removes individuals from the population. Adoption estimates were derived from a 2024 national survey of animal welfare organizations, indicating an average of 52,000 adoptions per year. Both processes were parameterized as constant annual rates but could be adjusted in future iterations to reflect seasonal or crisis-dependent variations. This parameter also includes a small proportion of cats that are euthanized for welfare reasons or die during surgery, which together represent less than 1% of treated individuals annually.

To prevent biologically implausible exponential growth and to reflect ecological constraints, a total carrying capacity (*K*) was applied in a second modeling phase. For each scenario, *K* was set at either 2.5× or 3× the initial population size, depending on the estimated ecological resilience of the territory. This value was applied globally across the entire metapopulation (i.e., sterilized and unsterilized individuals combined), acknowledging that both groups exert pressure on local resources such as shelter, food, and space.

A summary of the key demographic parameters and assumptions used in the Vortex model is provided in Supplementary Table S1, ensuring transparency and reproducibility of the simulation framework.

## 3. Results

### 3.1. National Estimate of Community Cats and Spatial Distribution by Region

The estimated population of community cats in Spain for 2024 was derived from administrative data submitted by 1128 municipalities, as described in Section 2.1. These data were stratified by population size to calculate cat-to-human and cat-to-colony ratios (Table 1), which were then applied to official population figures from the Spanish National Institute of Statistics [41,42] (INE, 2021) to extrapolate national estimates. This yielded a total of 1,812,241 cats distributed across 125,561 colonies, excluding Ceuta and Melilla. Colony sizes varied by municipality type, ranging from 15.68 cats per colony in municipalities with fewer than 500 inhabitants to 12.64 in those with over 50,000. The cat-to-human ratio showed a clear gradient, decreasing from 0.59 in the smallest municipalities to 0.01 in the largest urban centers.

**Figure 1:**
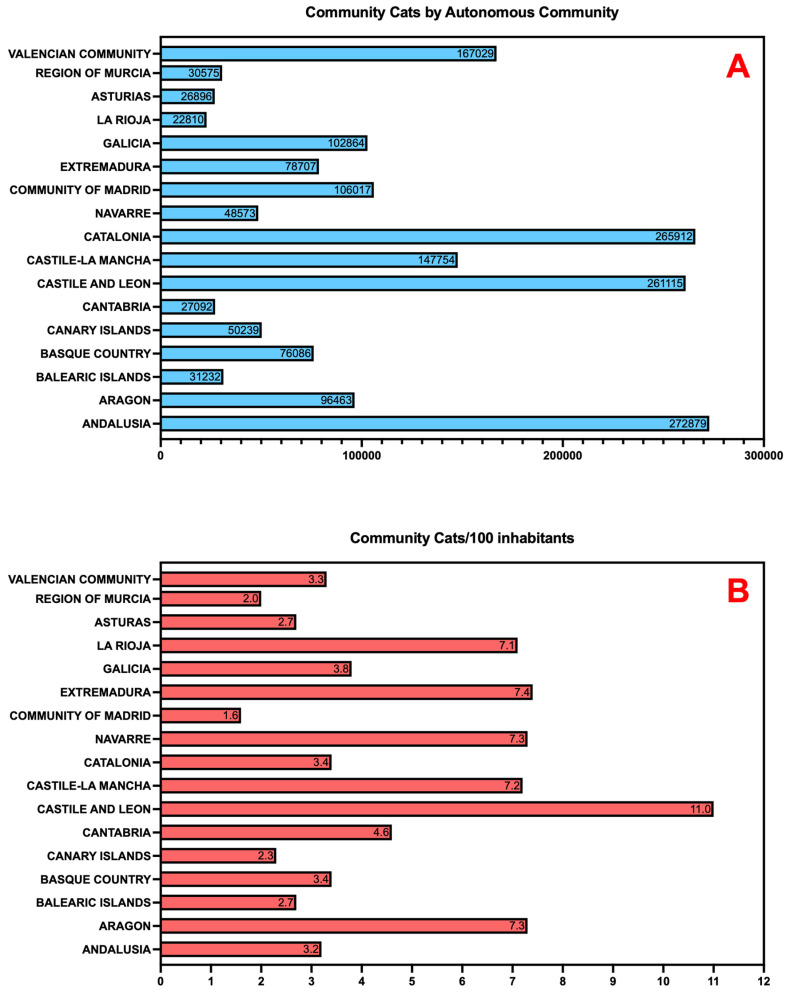
Estimated Distribution of Community Cats by Autonomous Community in Spain (2024). Panel (**A**) shows absolute population estimates per region. Panel (**B**) presents cat-to-human ratios, highlighting regional disparities in relative density.

Figure 1 illustrates the distribution of community cats by autonomous community. Panel A shows absolute numbers, with Andalusia, Catalonia, and Castile and Leon recording the highest populations (272,879; 265,912; and 261,115, respectively). Panel B presents cat-to-human ratios, highlighting relative prevalence. Castile and Leon (11.0 cats per 100 inhabitants), Extremadura (7.4), Aragon (7.3), Navarre (7.3), Castile-La Mancha (7.2), and La Rioja (7.1) exhibited the highest ratios, reflecting greater cat presence in predominantly rural regions, while Madrid (1.6), Catalonia (3.4), and Andalusia (3.2) showed lower relative densities despite large absolute populations (Figure 1B).

### 3.2. Reproductive Parameters and Viable Offspring Estimates

The reproductive output of community cats across Spain was modeled under stratified demographic and ecological scenarios, incorporating environmental constraints via the Reproductive Utilization Rate (RUR) and accounting for urban-rural differences in litter size. The simulations produced probability distributions for litters per female per year and kittens per litter across territorial contexts.

Table 2 summarizes the probability distribution of females producing 0 to 3 litters annually under four reproductive scenarios (low, medium, high, very high). As the RUR increased, the likelihood of three litters rose from 10.2% (low) to 37.7% (very high), while the proportion of non-reproductive females (0 litters) declined from 15.1% to 2.1%. Two litters per year remained the most common outcome across most scenarios, except in the low scenario, where one litter was slightly more frequent.

Table 3 presents probability distribution of litter sizes by urban and rural settings. Rural colonies were skewed toward larger litters, with 32.9% producing five kittens and 21.1% six. In contrast, urban colonies exhibited smaller litters, with 32.9% producing four kittens and only 5.8% producing six, reflecting environmental factors such as food availability, social stress, and habitat saturation.

These distributions were used to parameterize the annual reproductive yield per female in each scenario. Applying standardized mortality rates (65% for kittens under one year of age and 15% for adults) across scenarios ensured comparability and provided the foundation for projecting population trajectories under various sterilization strategies.

### 3.3. Baseline Population Structure and Projections Under Varying Sterilization Scenarios

The initial population structure for the simulation models was derived from the municipal survey data. Standardized cat-to-human ratios were extrapolated to all Spanish municipalities, stratified into eight demographic–environmental scenarios based on municipality size (urban or rural) and reproductive potential (low, medium, high, or very high), as detailed in Section 2.2. This produced eight typologies: UL, UM, UH, and UVH for urban areas, and RL, RM, RH, and RVH for rural ones.

Applying these ratios yielded a total estimated population of 1,813,939 community cats in 2024, distributed across eight subpopulations (Table 4). Rural areas concentrated 73.2% of the total (1,328,167 cats), while urban areas accounted for 26.8% (485,772 cats). This asymmetry reflects Spain’s demographic structure: over 70% of municipalities have fewer than 5000 inhabitants, despite accounting for less than 15% of the national human population. Limited intervention and higher reproductive potential in rural settings contribute to this concentration.

This baseline served to inform the population growth projections under various sterilization scenarios.

#### 3.3.1. Projection of the Cat Population Under Current Sterilization Rates (20% National Average)

The first projection simulated population dynamics under the current sterilization rate of approximately 20%, reflecting fragmented and discontinuous interventions that maintain the national 80:20 ratio between unsterilized and sterilized cats.

Under this baseline scenario, the national metapopulation increases sharply, saturating at approximately 2.5–3 fold the initial population (carrying capacity, *K*) by year 8 (2033). Although local dynamics vary—with rural low (RL) and medium (RM) reproductive potential areas stabilizing near K between years 15 and 25, due to ecological and social constraints—urban scenarios, particularly UL and UM, exhibit continued localized growth without fully reaching K, driven by reproductive dynamics and human subsidies.

**Figure 2:**
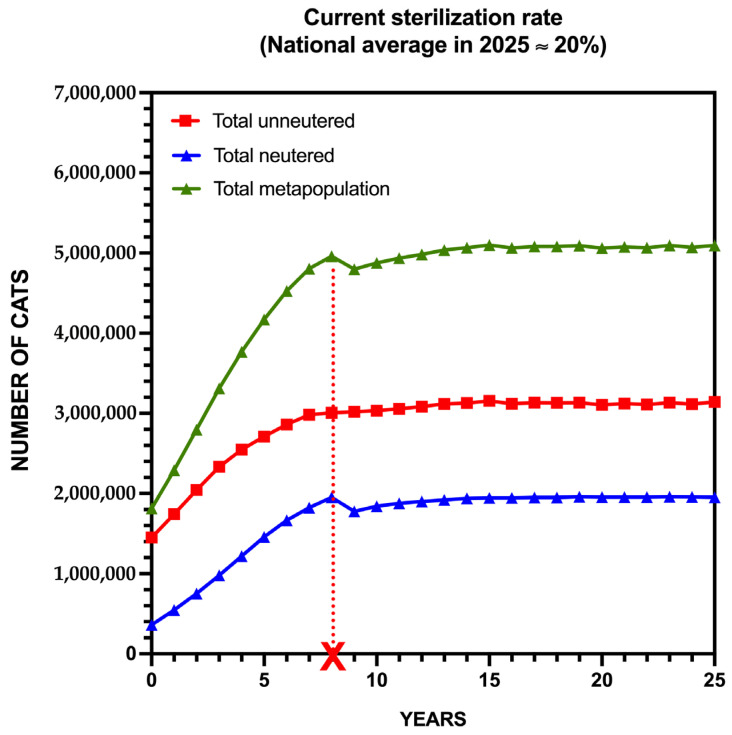
Projected Growth of The National Community Cat Population in Spain Under the Current Sterilization Scenario (20% coverage). The model reflects the continuation of fragmented efforts at current sterilization levels. The dotted red line indicates when carrying capacity (*K*) will be reached.

At the national level, the population rises from 1.8 million to over 4.6 million cats within the first decade, after which growth slows, stabilizing between 4.6 and 5 million cats, depending on reproductive potential and local *K* thresholds. Growth is primarily driven by the unsterilized subpopulation, while the sterilized fraction increases moderately, maintaining a relatively stable proportion (Figure 2).

These results indicate that current sterilization efforts are insufficient to prevent population growth, leading to the saturation of ecological and social carrying capacities in several rural scenarios, and to continued expansion in urban areas. This underscores the need for intensified and coordinated sterilization strategies, explored in subsequent projections.

#### 3.3.2. Projections Under Enhanced Sterilization Scenarios (40% and 80% Coverage)

To assess the impact of intensified sterilization, two alternative scenarios were modeled: 40% national coverage—doubling the current rate—and an idealized 80% coverage, widely cited in the literature as the threshold for effective for population control within a decade. While the 80% scenario illustrates theoretical efficacy, it remains unrealistic in many rural areas of Spain due to resource and logistical constraints.

Scenario 1: 40% National Sterilization Coverage

**Figure 3:**
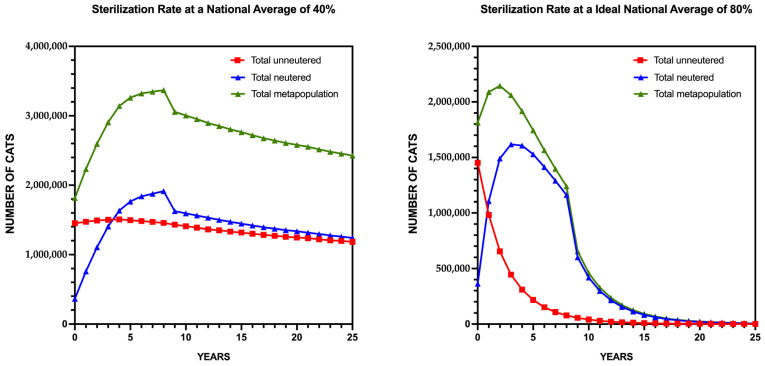
Projected Growth of the Community Cat population in Spain Under Two Enhanced Sterilization Scenarios: 40% (**Left Panel**) and 80% (**Right Panel**).

Under 40% coverage (Supplementary Table S3), population growth slows but continues, particularly in rural scenarios with higher reproductive potential. Nationally, the metapopulation rises from 1.8 million to over 3.4 million cats within 25 years, a notable reduction compared to the 20% baseline, but still insufficient for stabilization (Figure 3, Left Panel). The plateau effect is limited, appearing only in rural low (RL) and urban low (UL) contexts toward the end of the simulation, while growth persists in scenarios like rural very high (RVH) and urban medium (UM).

Scenario 2: 80% National Sterilization Coverage

In contrast, 80% coverage (Supplementary Table S4) achieves substantial population reduction across all scenarios. The national population declines sharply to fewer than 250,000 cats within 15 years, continuing to decrease thereafter (Figure 3, Right Panel). The unneutered subpopulation collapses, while sterilized individuals dominate, reflecting sustained reproductive suppression. Rural areas, with higher initial reproductive rates, exhibit faster declines, but all contexts approach functional extinction (i.e., minimal unneutered individuals) by the end of the simulation.

These contrasting scenarios highlight the sensitivity of population dynamics to sterilization intensity. While 40% coverage moderates growth, it fails to stabilize the population, whereas 80% coverage achieves sustained control, although it is unlikely to be feasible nationwide.

#### 3.3.3. Projections Under the Proposed National Strategy with Differentiated Sterilization Targets

To reflect Spain’s ecological, demographic, and administrative heterogeneity, a realistic intervention scenario was modeled, aligned with the PACF. This strategy integrates differentiated sterilization targets based on reproductive pressures and implementation feasibility.

The model applies 50% sterilization coverage in most territorial scenarios, increasing to 60% in urban areas with very high reproductive potential (UMA) and rural areas with high reproductive potential (RA). In rural areas with very high reproductive potential (RMA), where reproductive rates and ecological sensitivity are highest, coverage reaches 70%. This strategy prioritizes resource allocation toward regions with greater reproductive pressure while maintaining achievable targets elsewhere.

**Figure 4:**
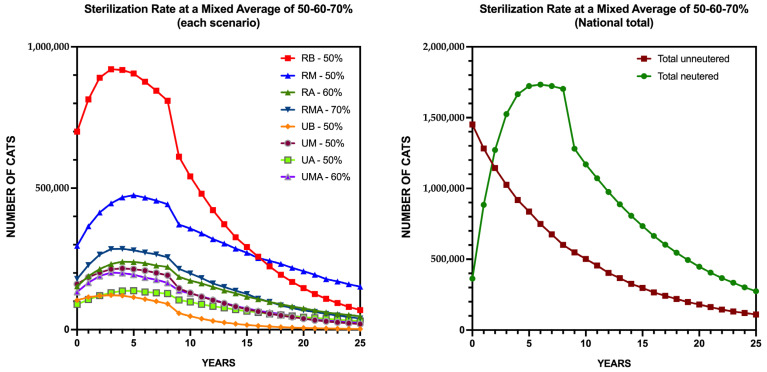
Projected Evolution of the Community Cat Population in Spain Under the Differentiated Sterilization Strategy. (**Left Panel**): Population trajectories across eight scenarios (UL, UM, UH, UVH, RL, RM, RH, and RVH) over 25 years, with sterilization rates of 50%, 60%, or 70%. (**Right Panel**): National totals.

Figure 4 illustrates these outcomes. In the Left Panel, population trajectories across the eight scenarios show steady declines in rural low (RL) and urban low (UL) contexts, stabilizing within 15–20 years. In higher reproductive scenarios (RA, UMA, RMA), population growth is substantially curtailed, with stabilization or slow declines achieved in the latter half of the period. The Right Panel presents the national totals, showing a steady decline in unsterilized individuals, while the sterilized population becomes predominant. This demographic shift progressively suppresses reproductive potential, stabilizing the population and reducing growth capacity without requiring complete reduction.

Supplementary Table S5 details projections for all eight scenarios. The Rural Very High Reproductive Potential (RMA) scenario shows the most significant reductions due to the higher sterilization target, while urban medium (UM) exhibits slower declines, reflecting complex urban dynamics and human-subsidized factors.

This differentiated approach offers a pragmatic pathway to stabilize cat populations, balancing biological efficacy with economic and operational feasibility across Spain’s diverse contexts.

## 4. Discussion

### 4.1. Foundational Principles and Strategic Coherence of the Plan

The PACF provides a coordinated, evidence-based strategy grounded in legal, ethical, environmental, and public health principles. This integrated approach aligns with the international consensus that community cat management must balance animal welfare with biodiversity conservation and public health objectives [21,50]. The Plan adopts a multidimensional framework, recognizing the shared responsibility of public administrations—particularly local governments—civil society, and the veterinary sector for the humane and effective management of free-roaming cats.

This strategy responds directly to the mandates of Law 7/2023, which frames cat management within a model that safeguards welfare, promotes biodiversity, and ensures efficient public resource use [1,37]. Spain’s nationally standardized legal framework is rare among feline management policies, positioning the country as one of the few with enforceable state-level guidelines.

Beyond being a technical document, the PACF embodies a political and social commitment to overcome decades of fragmented, under-resourced interventions. Historically, Spanish cat colony management relied on scattered municipal initiatives, often driven by volunteer networks without institutional backing [30,32,51]. By consolidating these efforts, the PACF seeks to professionalize and stabilize interventions, ensuring continuity and alignment with broader conservation and public health goals.

Crucially, the Plan is underpinned by the present study, which provides a comprehensive situational assessment of feline management in Spain as of 2024, integrating data from administrative records, field estimates, and scientific literature. This integrative approach aligns with established frameworks in animal health decision-making, where resource allocation and disease control strategies increasingly rely on robust, data-driven methodologies [52]. Moreover, the application of stratified modeling combined with standardized data collection represents a significant methodological advance, ensuring a solid baseline for policy development. Evidence-based frameworks that incorporate both quantitative data and strategic decision-making models are essential for addressing complex health challenges involving multiple stakeholders [53]. However, this baseline is provisional, designed to evolve as new tools—such as digital monitoring, welfare assessments, and diagnostic technologies—are adopted. This ensures that the PACF remains flexible and adaptive, integrating emerging scientific knowledge and practical experience through an iterative learning process. Such an approach is aligned with established adaptive management frameworks, which emphasize the systematic incorporation of monitoring and feedback to refine management strategies over time [54,55,56].

By integrating legal mandates, scientific evidence, and institutional coordination, the PACF lays the groundwork for long-term, sustainable cat management in Spain. In this context, we define sustainable management as the implementation of a scalable and scientifically guided TNR strategy aimed at stabilizing and gradually reducing the number of free-roaming cats to levels that are ecologically compatible and socially acceptable. This goal requires maintaining adequate sterilization coverage over time, matched by long-term institutional support and community engagement, with the target of reaching an average density of one cat per 100 inhabitants or less across Spain. The balanced approach of the PACF, combining biological efficacy, social legitimacy, and operational feasibility, offers a structured model that contrasts with the fragmented and heterogeneous strategies currently observed across European countries [57], where TNR implementation often depends on local initiatives rather than on national coordination.

### 4.2. Population Estimates and Territorial Distribution

The estimated national population of over 1.8 million community cats represents the first comprehensive attempt to quantify this phenomenon in Spain using standardized data and stratified modeling. Derived from a representative sample of over 1100 municipalities and adjusted for population size, this estimate provides a robust foundation for regional comparisons and policy prioritization. The finely tuned stratification system, disaggregating small municipalities into specific size classes, captures spatial heterogeneity in cat populations—an essential feature in ecological studies where stratified frameworks are recognized for enhancing representativeness and improving the accuracy of monitoring and modeling efforts [58,59,60].

The results reveal a pronounced demographic gradient between urban and rural areas. While large cities hold higher absolute numbers of cats, driven by human density and subsidies (e.g., food availability), rural municipalities exhibit far higher cat-to-human ratios and larger colony sizes—a pattern consistently observed in previous studies comparing urban and rural cat populations [16,24,27]. Notably, over 73% of the estimated population (1,328,168 cats) resides in rural areas, highlighting the need to adapt TNR programs beyond urban settings—a point often overlooked in urban-centric management models [24,27].

In rural contexts, TNR implementation faces distinct logistical and social challenges. Unlike urban colonies, rural cat populations often consist of loosely associated individuals living on farms or dispersed across small settlements, with minimal or irregular contact with human caregivers. These cats may not form recognizable colonies and are rarely subject to veterinary care. Moreover, rural residents may not identify themselves as owners, yet often provide food or tolerate the presence of cats, creating a form of *de facto* coexistence. As such, rural adaptation of TNR requires flexible strategies, including awareness campaigns, mobile sterilization units, and inter-municipal cooperation—especially in sparsely populated areas with limited infrastructure. Notably, in severely depopulated areas, cat populations may self-limit due to reduced anthropogenic food sources, but targeted intervention remains essential in semi-rural or peri-urban zones where resources and ecological impacts persist.

These findings underscore the value of integrating ecological sensitivity and spatial context into management planning. By transcending absolute counts, the PACF supports a risk-informed approach, aligning intervention intensity with both population dynamics and ecological vulnerability—thus directing resources where they yield the greatest ecological and welfare benefits.

### 4.3. Reproductive Dynamics and Population Trajectories: Implications for Management

The reproductive output of community cats, modeled under stratified scenarios, highlights how key demographic parameters shape long-term population trajectories. Two central factors—Reproductive Utilization Rates (RUR) and age-specific mortality rates—determine the system’s resilience. The RUR, modulated by territorial and environmental conditions, exhibits a broad gradient in reproductive potential, with higher litter frequencies and larger litter sizes in rural, high-productivity areas [21,23,24].

Mortality rates were uniformly standardized at 65% for kittens and 15% for adults across all scenarios. These values, based on empirical data from both managed and unmanaged populations [48,49], ensure comparability across models while preventing underestimation of population resilience, especially in rural contexts where survival rates are highly variable due to environmental pressures, disease, and limited human intervention. This conservative baseline reflects the minimum expected mortality within unmanaged colonies and supports robust, precautionary projections.

Simulations incorporating these parameters show that even modest variations in reproductive rates generate substantial cumulative impacts [18,21,24]. Under current conditions, with national sterilization coverage around 20%, Spain’s community cat population is projected to nearly triple over 25 years, reaching ecological saturation by Year 8. Increasing sterilization coverage to 40% slows this trajectory but remains insufficient to stabilize populations, particularly in high-reproductive rural areas.

These findings align with broader evidence suggesting that sterilization rates above 60–70% are typically required to induce population decline [17,18]. The 80% sterilization scenario, widely recognized as the international benchmark, achieves rapid and sustained population control, driving community cat populations toward functional extinction across all modeled contexts [18,19]. However, achieving and maintaining such high coverage, especially in rural areas with dispersed colonies and logistical challenges, is largely unattainable within Spain’s current administrative and infrastructural landscape [30,51].

To balance biological efficacy with operational feasibility, the PACF proposes a differentiated sterilization strategy: 50% national coverage, increasing to 60% in higher-risk areas, and 70% in rural zones with very high reproductive potential. This approach does not pursue eradication but sufficiently suppresses reproductive capacity, inducing a sustained population decline. Nationally, this scenario reduces the community cat population from 1.8 million in 2024 to approximately 1.06 million by 2049—a 41% reduction—while progressively shifting the demographic balance toward sterilized individuals, who would comprise over 70% of the population by the end of the projection period.

This trajectory sharply contrasts with the current baseline scenario (20% sterilization coverage), where the national cat population nearly triples within the first eight years, reaching ecological saturation between 4.6 and 5 million cats. Notably, this saturation point is imposed by theoretical carrying capacity thresholds, suggesting that actual growth could be even higher in the absence of such ecological constraints.

Under the PACF model, the decline is particularly marked in rural areas with high reproductive potential, where populations that would otherwise experience exponential growth are significantly suppressed. For example, in rural low-potential zones (RL), populations decline by over 70%, from 700,000 to approximately 184,000 cats, while urban high-potential areas (UH) experience more moderate declines due to higher baseline densities and human subsidies, stabilizing at lower rates but still achieving substantial demographic shifts.

These findings underscore the importance of territorially adapted strategies that align intervention intensity with local reproductive dynamics and ecological pressures. By moving from unchecked growth—characterized by rapid expansion and saturation within a decade—to a managed, gradual decline, the PACF achieves substantial population reduction while ensuring operational feasibility. This differentiated approach not only optimizes resource allocation, prioritizing efforts in areas where reproductive potential and ecological risks are highest but also represents a significant advance in evidence-based management, integrating animal welfare, biodiversity conservation, and public health objectives into national policy frameworks [18,54].

### 4.4. Limitations

While this study provides the most comprehensive demographic and economic modeling framework for managing community cats in Spain, several limitations must be acknowledged.

First, data availability and representativeness pose significant challenges. Despite the large sample of 1128 municipalities (13.9% of the national total), medium and large municipalities are overrepresented, while smaller rural areas are underrepresented. Although stratified modeling mitigates some bias, localized discrepancies in population dynamics or management effectiveness may persist. Additionally, the absence of a national cat census prior to 2024 required reliance on administrative data from municipal subsidy applications, which, while standardized, only cover participating municipalities and may exclude areas with unmanaged colonies. Moreover, these data likely reflect two opposing biases: underreporting in areas with unregistered colonies, particularly in rural zones or urban peripheries, and overreporting by municipalities seeking to secure sufficient funding to manage future increases in colony size. This dual bias, inherent in reliance on self-reported administrative data, highlights the need for ongoing, field-based validation and national monitoring efforts.

Second, the use of Vortex software (Version 10.6.0.), originally designed for wildlife species with longer reproductive cycles, introduces methodological constraints. Vortex does not allow reproductive cycles shorter than one year or sub-annual time steps, limiting its alignment with feline biology. Adjustments, such as the Reproductive Utilization Rate (RUR), were applied to compensate, but may not fully capture urban-rural reproductive nuances or seasonal variability, particularly in Mediterranean climates with prolonged estrus periods.

Third, assumptions regarding reproductive parameters and mortality rates—though based on the best available literature—may not reflect local variability influenced by predation, disease, food availability, or human intervention. For instance, kitten mortality was standardized at 65% across scenarios, though empirical data suggest broader ranges. Similarly, abandonment and adoption rates were modeled as constants, despite potential fluctuations linked to economic conditions, cultural practices, or policy changes.

Fourth, the abandonment and adoption rates applied have been kept constant throughout the entire timeframe of the plan, as the evolution of these practices in the coming years cannot be predicted. However, an increase in adoption and a decrease in abandonment are anticipated, due to the public policies currently being implemented to achieve these goals.

Finally, Spain’s environmental and social heterogeneity suggests that a uniform application of sterilization targets (e.g., the 50–60–70% model) may yield variable outcomes. Regions with fragmented municipal structures, limited veterinary infrastructure, or heightened ecological sensitivity (e.g., islands or protected areas) may require adaptive management strategies beyond the current model. Future iterations should incorporate real-time data from national monitoring platforms and field assessments to refine projections and resource allocation.

Despite these limitations, this framework offers a robust foundation for evidence-based policy design, providing essential insights for national-scale population management. Continued data collection, technological refinement, and adaptive governance will be essential for improving future projections and ensuring effective implementation. While this framework identifies TNR as the most viable and legally mandated strategy for community cat management in Spain, ecological outcomes—particularly in sensitive areas—require continuous evaluation. Monitoring efforts should aim to refine and adapt TNR implementation itself, enhancing its effectiveness and alignment with biodiversity conservation, without deviating from its ethical foundation. This reinforces the need for an open, evidence-based approach that integrates animal welfare goals with ecological integrity, ensuring that management interventions remain responsive to emerging challenges and conservation priorities.

## 5. Conclusions

This study presents the first comprehensive national framework for the ethical management of community cat populations in Spain, integrating legal mandates, stratified demographic modeling, and territorial adaptation to reproductive potential. The proposed strategy—structured around differentiated sterilization targets (50–60–70%)—balances biological efficacy with operational feasibility, addressing the ecological and demographic diversity of the Spanish territory.

Population projections based on standardized municipal data and scenario-specific reproductive modeling indicate that, while high sterilization rates (80%) lead to the most rapid and sustained reductions, a more realistic, territory-sensitive strategy can achieve population stabilization and long-term decline, particularly in areas with high reproductive potential.

Despite limitations related to data representativeness, reproductive parameter variability, and modeling assumptions, the simulation framework provides a robust foundation for informed planning and resource allocation. Its design supports adaptive management through stratified monitoring and feedback mechanisms and can guide the phased implementation of national objectives established by Law 7/2023.

While this study did not assess ecological impacts directly, the proposed framework is designed to be compatible with biodiversity conservation goals. By reducing population growth through non-lethal methods and enabling context-specific planning, it provides a foundation for integrating animal welfare with broader urban and environmental objectives under a One Health perspective.

Finally, while the approach is grounded in the Spanish context, the methodology—especially the integration of legal, ecological, and demographic dimensions—may serve as a reference for other countries exploring ethical and sustainable community cat management within broader urban and landscape planning efforts.

## Figures and Tables

**Table 1:** Typology of Municipalities by Population Size: Sample Representation and Cat-to-Human and Cat-to-Colony Ratios.

Typology by Population Size	Number of Municipalities in the Sample	Total Number of Municipalities ^a^	Percentage of Municipalities ^a^	Cat-to-Human Ratio	Cat-to-Colony Ratio
<500 inhabitants	109	3.977	2.74%	0.59	15.68
500–5.000 inhabitants	477	2.833	16.83%	0.12	15.05
5.000–10.000 inhabitants	186	549	33.87%	0.04	13.29
10.000–50.000 inhabitants	278	611	45.49%	0.03	14.14
>50.000 inhabitants	78	151	51.65%	0.01	12.64

^a^ Source: National Statistics Institute of Spain (INE), Population by Provinces and Municipality Size, 2025. https://www.ine.es/jaxiT3/Tabla.htm?t=2917&L=0 (accessed on 7 May 2025).

**Table 2:** Probability Distribution of Litters per Reproductive Female per Year, Stratified by Scenario.

RUR	Scenario	0 Litters (%)	1 Litter (%)	2 Litters (%)	3 Litters (%)
0.55	Low (L)	15.1	39.77	34.91	10.22
0.65	Medium (M)	8.96	33.19	40.98	16.87
0.75	High (H)	4.76	25.13	44.2	25.91
0.85	Very High (VH)	2.14	16.69	43.46	37.71

**Table 3:** Probability Distribution of Kittens per Litter in Urban and Rural Environments.

Number of Kittens per Litter	Urban (%)	Rural (%)
1	2.43	0.52
2	11.62	3.58
3	27.67	13.54
4	32.92	28.37
5	19.56	32.89
6	5.80	21.10

**Table 4:** Baseline Community Cat Population in Spain (2024), Stratified by Scenario.

Scenario	Municipality Type	Reproductive Potential	Initial Population (n)
UL	Urban	Low (L)	103,676
UM	Urban	Medium (M)	160,648
UH	Urban	High (H)	88,719
UVH	Urban	Very High (VH)	132,726
RL	Rural	Low (L)	700,056
RM	Rural	Medium (M)	295,906
RH	Rural	High (H)	153,003
RVH	Rural	Very High (VH)	179,202
Urban Total	-	-	485,769
Rural Total	-	-	1,328,167
National Total	-	-	1,813,936

## Data Availability

All relevant data have been included either in the main manuscript or Supplementary Materials. Additional data can be obtained by direct request to the corresponding author.

## Supplementary Materials

The following supporting information can be downloaded at: https://www.mdpi.com/article/10.3390/ani15152278/s1, Figure S1: Classification of Spanish provinces into four reproductive intensity levels (Low, Medium, High, Very High) based on daylight hours and winter temperatures, which served to define eight demographic-environmental scenarios for simulation; Table S1: Key demographic parameters and assumptions used in the Vortex population model for community cats in Spain.; Table S2: Projected evolution of the community cat population in Spain under a baseline 20% sterilization scenario (2024–2049); Table S3: Projected evolution of the community cat population in Spain under a 40% sterilization scenario (2024–2049); Table S4: Projected evolution of the community cat population in Spain under an ideal (but unrealistic) 80% sterilization scenario (2024–2049); Table S5: Projected evolution of the community cat population in Spain under a realistic sterilization scenario (2024–2049), assuming 50% coverage overall, 60% in UMA and RA scenarios, and 70% in RMA scenarios.
