# Supplementary material for: Territorially Stratified Modeling for Sustainable Management of Free-Roaming Cat Populations in Spain: A National Approach to Urban and Rural Environmental Planning
Source: Animals (Basel). 2025 Aug 4;15(15):2278. doi: 10.3390/ani15152278 (PMC12345437; doi:10.3390/ani15152278)
Supplement: Supplementary file 1 [file animals-15-02278-s001.zip › Supplementary Table S5.pdf]

Supplementary Table S5. Projected evolution of the community cat population in Spain under a realistic sterilization scenario (2024–2049), assuming 50% coverage overall, 60% in UMA and RA scenarios, and 70% in RMA scenarios.

Populations	Year 0	Year 1	Year 2	Year 3	Year 4	Year 5	Year 6	Year 7	Year 8	Year 9	Year 10	Year 11	Year 12	Year 13	Year 14	Year 15	Year 16	Year 17	Year 18	Year 19	Year 20	Year 21	Year 22	Year 23	Year 24	Year 25
RL unneutered	560045	484918	423488	366142	312162	280175	248002	217894	193418	174459	150617	132125	114462	98740	84849	78874	71027	60680	51829	45016	37768	32173	27861	22869	19943	17987
RL neutered	140011	328891	467359	554681	605925	625475	628630	626677	616025	436985	391319	348309	307908	273738	241494	212596	186309	162812	141794	123599	108631	93627	81156	70970	60005	51092
RL metapopulation	700056	813809	890847	920824	918087	905650	876632	844571	809442	611444	541936	480434	422370	372478	326343	291470	257336	223492	193622	168615	146399	125800	109016	93839	79948	69079
RM unneutered	236724	226088	208161	194192	184446	176080	164283	153644	138686	127829	128325	121586	111493	106382	98779	93290	84311	82278	77654	72511	69378	64929	57663	56060	52790	49034
RM neutered	59181	138858	205857	251882	283111	298980	302483	302483	304260	243706	228907	218386	208315	198098	187392	178210	168609	161094	154256	145563	136744	129004	120702	114212	107464	102479
RM metapopulation	295905	364946	414018	446074	467558	475060	466766	456127	442947	371534	357232	339972	319807	304480	286171	271500	252919	243371	231910	218074	206122	193933	178365	170272	160254	151513
RH unneutered	122402	109491	98428	90273	85470	79576	73114	64811	61285	56509	51126	48509	44117	39784	36604	30859	28790	25712	23215	21391	20421	17001	15614	14326	14197	12222
RH neutered	30600	79371	115690	141366	154570	159454	161487	161440	160520	130079	121974	114841	106509	98219	91734	84935	78727	71997	66174	60326	54652	50042	45268	40746	37630	34375
RH metapopulation	153002	188862	214118	231638	240040	239030	234601	226251	221805	186587	173101	163350	150626	138003	128338	115794	107517	97709	89389	81717	75072	67044	60882	55072	51828	46597
RVH unneutered	143361	125852	115421	105139	95684	87261	79260	75548	66937	61321	56936	49696	41113	39082	35047	32139	25657	23336	19685	17396	15523	14465	12530	11175	9670	8234
RVH neutered	35840	101805	149771	179468	189728	193125	193063	190438	188820	153311	141569	132609	121754	111370	102382	93496	83545	74506	65827	58410	52424	47676	42638	38902	34874	30812
RVH metapopulation	179201	227657	265193	284607	285413	280386	272322	265987	255757	214632	198505	182305	162867	150452	137428	125635	109202	97841	85512	75806	67947	62141	55169	50078	44544	39046
UL unneutered	82941	65640	53035	44855	36573	29293	23813	19618	15468	12636	10328	8178	6452	5470	4468	3634	2978	2378	1956	1609	1340	1127	906	740	626	477
UL neutered	20735	48912	66427	76877	83375	84753	83576	80076	75438	44881	36817	30273	24112	19516	15819	12855	10303	8405	6819	5610	4627	3938	3227	2611	2131	1737
UL metapopulation	103676	114553	119463	121732	119948	114046	107389	99694	90906	57517	47145	38451	30565	24986	20288	16488	13281	10782	8775	7219	5967	5065	4133	3351	2756	2214
UM unneutered	128518	108940	96402	86934	75971	67078	59836	53023	46271	41915	36244	33182	30342	27075	22839	19905	18352	15343	14322	12353	10449	8574	7708	6841	6011	5249
UM neutered	32192	75723	105427	126543	140042	146687	148365	147379	145626	103710	93124	82486	73548	65634	58138	51465	45487	40287	35401	31793	28038	24582	21383	18720	16373	14182
UM metapopulation	160710	184663	201830	213477	216012	213765	208201	200402	191896	145624	129368	115668	103890	92709	80977	71370	63840	55630	49722	44146	38486	33156	29091	25561	22383	19431
UH unneutered	70975	64663	60568	57366	53232	49097	42643	38338	36448	33561	31504	28252	26258	25756	23275	20546	19056	17049	15952	15070	13658	13677	12699	11279	10539	10797
UH neutered	17743	41476	59289	73134	83064	88207	90366	91611	90679	70983	66073	60992	55701	50979	47277	44116	41345	38139	35433	32663	29996	27755	25580	23737	21819	19891
UH metapopulation	88718	106139	119857	130500	136296	137303	133009	129948	127127	104544	97577	89244	81959	76735	70551	64661	60401	55188	51385	47733	43654	41433	38279	35016	32358	30688
UVH unneutered	106181	96508	88153	80808	74203	67928	58330	52906	43280	40471	37239	33676	28975	24181	20425	18419	15815	15002	14224	12623	11592	10241	9253	7679	6671	5680
UVH neutered	26545	69163	101250	120533	125457	126412	125311	123251	121563	96840	90272	83511	76959	69770	62709	56361	50281	45496	40419	36339	32154	29020	26496	23939	21546	19306
UVH metapopulation	132726	165672	189404	201341	199661	194340	183642	176157	164843	137312	127512	117188	105935	93951	83133	74780	66096	60498	54643	48963	43746	39261	35749	31619	28217	24986

[a] Abbreviations:
RL, RM, RH, RVH = rural municipalities with low, medium, high, or very high reproductive potential;
UL, UM, UH, UVH = urban municipalities with low, medium, high, or very high reproductive potential.
