# Supplementary material for: Territorially Stratified Modeling for Sustainable Management of Free-Roaming Cat Populations in Spain: A National Approach to Urban and Rural Environmental Planning
Source: Animals (Basel). 2025 Aug 4;15(15):2278. doi: 10.3390/ani15152278 (PMC12345437; doi:10.3390/ani15152278)
Supplement: Supplementary file 1 [file animals-15-02278-s001.zip › Supplementary Table S1.pdf]

Supplementary Table S1. Key demographic parameters and assumptions used in the Vortex population model for community cats in Spain.

Parameter	Value/Assumption	Justification
**Carrying Capacity (K)**	2.5× or 3× initial population (scenario-dependent)	Reflects ecological resilience and prevents unrealistic exponential growth.
**Reproductive Rate**	Up to 3 litters/year per breeding female	Based on biological potential, adjusted by Reproductive Utilization Rate (RUR).
**Litter Size (mean ± SD)**	Urban: 3.75 ± 1.2 kittens; Rural: 4.75 ± 1.3 kittens	Derived from field studies and literature on urban vs. rural populations.
**Kitten Mortality (<1 year)**	65%	Conservative estimate based on unmanaged colony data (Grieco et al., 2021; Dolan et al., 2021).
**Adult Mortality (>1 year)**	15%	Standardized across scenarios for comparability; aligns with feral cat studies.
**Reproductive Lifespan (both sexes)**	1 to 8 years	Conservatively set due to Vortex's annual timestep limitations; avoids underestimation of long-term reproductive contribution.
**Sterilization Impact**	100% reproductive suppression in sterilized individuals	Modeled via Vortex "dispersal" function to dynamically transfer to sterile pool.
**Disease Outbreaks**	5% annual probability; 30% increased mortality if triggered	Models stochastic catastrophic events (e.g., panleukopenia, poisoning).
**Abandonment Rate**	109,000 cats/year, distributed across 8 scenarios (RL, RM, RH, etc.)	Based on 2024 national survey of 500 municipalities (DGDA data); proportional allocation by scenario.
**Adoption and Removal Rate ("Harvest")**	52,000 cats/year, plus 1% additional removals (euthanasia or perioperative death), distributed across 8 scenarios (RL, RM, RH, etc.)	Derived from national survey of animal welfare organizations (2024); ; includes humane euthanasia and surgical mortality based on expert estimates; proportional allocation by scenario.
