# Supplementary material for: Territorially Stratified Modeling for Sustainable Management of Free-Roaming Cat Populations in Spain: A National Approach to Urban and Rural Environmental Planning
Source: Animals (Basel). 2025 Aug 4;15(15):2278. doi: 10.3390/ani15152278 (PMC12345437; doi:10.3390/ani15152278)
Supplement: Supplementary file 1 [file animals-15-02278-s001.zip › Supplementary Table S2.pdf]

Supplementary Table S2. Projected evolution of the community cat population in Spain under a baseline 20% sterilization scenario (2024–2049).

Populations [a]	Year 0	Year 1	Year 2	Year 3	Year 4	Year 5	Year 6	Year 7	Year 8	Year 9	Year 10	Year 11	Year 12	Year 13	Year 14	Year 15	Year 16	Year 17	Year 18	Year 19	Year 20	Year 21	Year 22	Year 23	Year 24	Year 25
**RL unneutered**	560045	633285	713091	798773	851526	927197	977758	1055512	1065600	1064935	1044225	1054256	1064071	1089890	1082742	1099080	1078922	1089785	1096037	1101681	1091707	1104304	1103519	1123715	1094417	1124490
**RL neutered**	140011	211959	285342	358973	437001	514192	589088	656173	716705	634641	662445	680317	685598	696208	702489	704788	700148	701597	699641	707134	707927	712573	714228	718050	719792	718317
**RL metapopulation**	700056	845244	998433	1157745	1288526	1441388	1566846	1711685	1782304	1699575	1706670	1734573	1749670	1786098	1785231	1803868	1779070	1791382	1795678	1808816	1799634	1816876	1817747	1841765	1814210	1842807
**RM unneutered**	236724	287359	336527	383198	426528	445995	483443	498419	495679	493255	504323	506802	521076	525139	521141	529872	530605	527793	532522	511402	511176	510581	513177	513532	522592	517924
**RM neutered**	59181	88756	122396	161252	202182	242201	277005	305742	326508	299289	310460	316928	319054	322572	329297	331348	332424	335071	336961	338643	335875	332803	332176	333603	329763	326488
**RM metapopulation**	295905	376115	458923	544450	628710	688196	760447	804161	822187	792544	814783	823731	840130	847711	850438	861220	863029	862864	869483	850044	847051	843384	845353	847136	852355	844412
**RH unneutered**	122402	172210	219774	247103	272300	278931	280301	281693	277959	277419	278781	280530	279153	279324	277777	282872	279804	283865	277838	281708	273239	278438	278273	278276	280024	279442
**RH neutered**	30600	46306	67559	93235	119811	147432	166380	176403	182934	171982	174897	175258	176706	176103	176543	176557	177048	177731	177708	176482	176098	175906	175680	176304	176080	176506
**RH metapopulation**	153002	218516	287333	340337	392111	426363	446681	458095	460893	449401	453678	455788	455859	455427	454320	459429	456852	461596	455546	458190	449337	454344	453953	454579	456104	455947
**RVH unneutered**	143361	199012	243627	290118	313960	323908	332422	329808	337777	338254	340837	341348	333266	330040	335656	335256	332284	335632	332914	339351	337793	338101	331928	328503	331579	331754
**RVH neutered**	35840	54141	78432	107612	139294	169298	193534	205789	214264	203080	208282	210152	211177	211097	211447	210739	212031	210186	210226	210198	209753	209666	210628	209316	209428	210222
**RVH metapopulation**	179201	253153	322059	397731	453254	493206	525957	535597	552041	541334	549118	551500	544443	541137	547103	545995	544315	545819	543140	549549	547546	547767	542556	537819	541007	541976
**UL unneutered**	82941	87348	93514	102921	111791	115016	121078	123601	129715	126797	132760	136514	138960	138841	139767	140730	142145	144803	141300	144420	141612	143829	141660	138432	136911	133463
**UL neutered**	20735	31384	41256	50953	61015	70403	78937	85732	92170	76088	78887	81974	84183	86259	87579	88629	89982	90422	91621	90730	90685	90528	90983	91380	90761	89273
**UL metapopulation**	103676	118732	134770	153874	172806	185419	200015	209332	221884	202885	211647	218487	223143	225100	227346	229358	232128	235225	232921	235150	232297	234357	232643	229812	227672	222736
**UM unneutered**	128518	145513	165741	178252	198135	215287	234690	241144	253241	259538	266487	272797	280784	285740	291444	292874	287850	285860	282205	279943	275767	278254	278611	282114	283956	284578
**UM neutered**	32192	48872	65503	82828	100103	117523	134671	149068	162307	145294	153102	158227	164667	169006	171083	170731	171638	172753	171964	173507	173206	171579	170772	170772	170204	170202
**UM metapopulation**	160710	194385	231245	261081	298238	332811	369360	390212	415548	404832	419589	431024	445451	454746	462527	463605	459489	458613	454169	453450	448973	449833	449383	452887	454160	454780
**UH unneutered**	70975	84102	104083	123572	137402	148959	159605	170005	171455	174840	176931	176296	178983	179790	187250	180189	179103	180916	178080	181253	182029	184096	182181	179497	182613	180520
**UH neutered**	17743	26704	36664	48523	62208	75354	86733	94583	100926	95065	97880	99509	100382	101146	102383	102560	102921	102978	103328	103559	103720	103666	103257	102882	102892	103408
**UH metapopulation**	88718	110806	140747	172095	199610	224313	246337	264587	272381	269904	274811	275804	279364	280935	289633	282749	282023	283894	281408	284813	285748	287762	285438	282380	285505	283928
**UVH unneutered**	106181	134465	167643	209055	235538	255526	271660	281560	276991	285173	290717	288581	286907	288897	291936	293366	290381	283916	290536	293459	293554	284938	281457	289570	283969	289752
**UVH neutered**	26545	39939	56153	75332	98510	122731	139576	149837	157147	151108	155809	157191	158026	157970	158181	158892	158335	158950	158168	158717	158615	158737	158191	158032	157715	158073
**UVH metapopulation**	132726	174404	223795	284387	334048	378257	411236	431397	434138	436281	446527	445771	444933	446867	450117	452258	448716	442866	448705	452176	452170	443674	439648	447602	441684	447825

[a] Abbreviations:
RL, RM, RH, RVH = rural municipalities with low, medium, high, or very high reproductive potential;
UL, UM, UH, UVH = urban municipalities with low, medium, high, or very high reproductive potential.
