# Supplementary material for: Territorially Stratified Modeling for Sustainable Management of Free-Roaming Cat Populations in Spain: A National Approach to Urban and Rural Environmental Planning
Source: Animals (Basel). 2025 Aug 4;15(15):2278. doi: 10.3390/ani15152278 (PMC12345437; doi:10.3390/ani15152278)
Supplement: Supplementary file 1 [file animals-15-02278-s001.zip › Supplementary Table S3.pdf]

**Supplementary Table S3.** Projected evolution of the community cat population in Spain under a 40% sterilization scenario (2024–2049).

| Populations | Year 0 | Year 1 | Year 2 | Year 3 | Year 4 | Year 5 | Year 6 | Year 7 | Year 8 | Year 9 | Year 10 | Year 11 | Year 12 | Year 13 | Year 14 | Year 15 | Year 16 | Year 17 | Year 18 | Year 19 | Year 20 | Year 21 | Year 22 | Year 23 | Year 24 | Year 25 |
|---|---|---|---|---|---|---|---|---|---|---|---|---|---|---|---|---|---|---|---|---|---|---|---|---|---|---|
| RL unneutered | 560045 | 538404 | 515173 | 494474 | 476803 | 457027 | 432874 | 417749 | 399803 | 378494 | 362835 | 345821 | 330527 | 320758 | 307642 | 297046 | 284720 | 274370 | 269454 | 258684 | 249356 | 240270 | 226489 | 218145 | 209660 | 201325 |
| RL neutered | 140011 | 292614 | 417507 | 516628 | 593906 | 642447 | 671739 | 686196 | 696706 | 552975 | 531337 | 511536 | 492549 | 472333 | 454616 | 437759 | 421537 | 405949 | 391989 | 379197 | 367890 | 354591 | 340089 | 325845 | 313549 | 302020 |
| RL metapopulation | 700056 | 831018 | 932680 | 1011101 | 1070709 | 1099475 | 1104613 | 1103945 | 1096509 | 931469 | 894173 | 857357 | 823076 | 793090 | 762259 | 734805 | 706257 | 680320 | 661443 | 637880 | 617246 | 594861 | 566577 | 543990 | 523209 | 503345 |
| RM unneutered | 236724 | 244021 | 251807 | 254264 | 255861 | 255736 | 256320 | 258679 | 259329 | 257099 | 253461 | 253671 | 250879 | 252704 | 251920 | 247664 | 246139 | 244078 | 245698 | 242643 | 241808 | 241993 | 242051 | 241771 | 242671 | 239461 |
| RM neutered | 59181 | 124056 | 181771 | 233467 | 273563 | 296952 | 311707 | 319233 | 327548 | 288364 | 287835 | 285678 | 283743 | 281885 | 280462 | 278797 | 276501 | 273704 | 272557 | 271901 | 270583 | 269632 | 268887 | 268213 | 267283 | 266741 |
| RM metapopulation | 295905 | 368076 | 433577 | 487731 | 529424 | 552688 | 568027 | 577912 | 586878 | 545463 | 541296 | 539349 | 534622 | 534589 | 532382 | 526461 | 522640 | 517783 | 518255 | 514544 | 512391 | 511625 | 510939 | 509984 | 509955 | 506202 |
| RH unneutered | 122402 | 134259 | 146996 | 157240 | 166823 | 172212 | 178167 | 180783 | 183296 | 185163 | 187167 | 188086 | 188385 | 188906 | 188751 | 191650 | 191741 | 190484 | 188897 | 191696 | 191786 | 195125 | 196190 | 192097 | 192582 | 191751 |
| RH neutered | 30600 | 63942 | 96282 | 127562 | 151234 | 163788 | 170594 | 174010 | 179660 | 167871 | 169408 | 170306 | 171027 | 171539 | 171415 | 171281 | 171188 | 171237 | 171123 | 171724 | 172186 | 172472 | 173001 | 173257 | 173142 | 173212 |
| RH metapopulation | 153002 | 198201 | 243277 | 284801 | 318057 | 336000 | 348761 | 354793 | 362956 | 353034 | 356575 | 358392 | 359412 | 360445 | 360166 | 362931 | 362929 | 361720 | 360020 | 363421 | 363972 | 367597 | 369190 | 365355 | 365724 | 364963 |
| RVH unneutered | 143361 | 171873 | 196801 | 216512 | 230516 | 241406 | 248382 | 253940 | 257912 | 263900 | 267841 | 270886 | 270466 | 268709 | 268356 | 271206 | 270396 | 269593 | 268609 | 270165 | 274564 | 274173 | 273677 | 272971 | 275817 | 277017 |
| RVH neutered | 35840 | 74933 | 117381 | 160364 | 188517 | 201567 | 207541 | 210526 | 216789 | 208295 | 209754 | 210796 | 211364 | 211672 | 211784 | 211776 | 211882 | 212101 | 211952 | 212261 | 212469 | 212854 | 213158 | 213358 | 213450 | 213868 |
| RVH metapopulation | 179201 | 246806 | 314181 | 376876 | 419033 | 442974 | 455923 | 464466 | 474701 | 472195 | 477595 | 481682 | 481830 | 480381 | 480140 | 482982 | 482278 | 481694 | 480561 | 482426 | 487033 | 487027 | 486835 | 486329 | 489267 | 490885 |
| UL unneutered | 82941 | 73509 | 65763 | 59144 | 53042 | 47553 | 43315 | 39254 | 35428 | 31615 | 28240 | 25108 | 22462 | 19967 | 18047 | 16125 | 14383 | 12779 | 11563 | 10214 | 9248 | 8397 | 7600 | 6832 | 6241 | 5631 |
| UL neutered | 20735 | 43345 | 59801 | 71627 | 79627 | 84262 | 86614 | 86990 | 86313 | 59608 | 54169 | 48720 | 43966 | 39464 | 35391 | 31668 | 28339 | 25347 | 22670 | 20358 | 18194 | 16378 | 14728 | 13220 | 11914 | 10712 |
| UL metapopulation | 103676 | 116855 | 125564 | 130772 | 132669 | 131815 | 129929 | 126243 | 121741 | 91222 | 82409 | 73827 | 66428 | 59432 | 53438 | 47793 | 42722 | 38126 | 34234 | 30572 | 27442 | 24774 | 22328 | 20052 | 18155 | 16343 |
| UM unneutered | 128518 | 122459 | 116539 | 111221 | 105350 | 100305 | 95725 | 90984 | 86451 | 82006 | 77723 | 73905 | 70567 | 67565 | 64202 | 61177 | 58546 | 56044 | 53816 | 51367 | 49025 | 47217 | 44784 | 42762 | 40800 | 39519 |
| UM neutered | 32192 | 67106 | 95302 | 117548 | 134621 | 145979 | 152266 | 154746 | 156562 | 122473 | 116906 | 111522 | 106475 | 101678 | 97371 | 92977 | 88752 | 84970 | 81558 | 78200 | 75058 | 71742 | 68458 | 65348 | 62635 | 59911 |
| UM metapopulation | 160710 | 189565 | 211841 | 228769 | 239971 | 246283 | 247990 | 245730 | 243013 | 204479 | 194629 | 185427 | 177043 | 169243 | 161573 | 154154 | 147298 | 141014 | 135374 | 129566 | 124083 | 118958 | 113243 | 108110 | 103434 | 99430 |
| UH unneutered | 70975 | 72873 | 73605 | 74796 | 74955 | 74793 | 75246 | 75681 | 75880 | 74205 | 73570 | 72617 | 71474 | 71743 | 71468 | 71250 | 71525 | 69884 | 68584 | 67763 | 67174 | 66505 | 66312 | 65791 | 65732 | 64974 |
| UH neutered | 17743 | 37119 | 54361 | 69344 | 81151 | 88287 | 92468 | 94812 | 97242 | 84306 | 83770 | 82890 | 81995 | 81322 | 80534 | 79834 | 79070 | 78516 | 77997 | 77326 | 76726 | 76100 | 75266 | 74528 | 73578 | 72817 |
| UH metapopulation | 88718 | 109992 | 127965 | 144140 | 156106 | 163080 | 167714 | 170493 | 173122 | 158512 | 157340 | 155508 | 153469 | 153065 | 152002 | 151084 | 150594 | 148400 | 146581 | 145089 | 143900 | 142605 | 141578 | 140319 | 139311 | 137792 |
| UVH unneutered | 106181 | 115538 | 124334 | 133147 | 142856 | 147706 | 152224 | 154375 | 157287 | 158662 | 157917 | 158742 | 158335 | 160194 | 160950 | 161981 | 163605 | 166062 | 163739 | 163505 | 162606 | 163866 | 162525 | 164781 | 163564 | 163972 |
| UVH neutered | 26545 | 55457 | 83301 | 109659 | 130462 | 141609 | 147075 | 149674 | 153689 | 141364 | 141704 | 141646 | 141509 | 141450 | 141929 | 142063 | 142168 | 141981 | 142195 | 142205 | 142470 | 142656 | 142671 | 142601 | 142584 | 142169 |
| UVH metapopulation | 132726 | 170995 | 207636 | 242806 | 273318 | 289315 | 299299 | 304050 | 310977 | 300026 | 299621 | 300389 | 299844 | 301644 | 302879 | 304044 | 305773 | 308044 | 305934 | 305710 | 305075 | 306522 | 305196 | 307382 | 306148 | 306141 |

[a] Abbreviations:
RL, RM, RH, RVH = rural municipalities with low, medium, high, or very high reproductive potential;
UL, UM, UH, UVH = urban municipalities with low, medium, high, or very high reproductive potential.
