# Supplementary material for: Territorially Stratified Modeling for Sustainable Management of Free-Roaming Cat Populations in Spain: A National Approach to Urban and Rural Environmental Planning
Source: Animals (Basel). 2025 Aug 4;15(15):2278. doi: 10.3390/ani15152278 (PMC12345437; doi:10.3390/ani15152278)
Supplement: Supplementary file 1 [file animals-15-02278-s001.zip › Supplementary Table S4.pdf]

Supplementary Table S4. Projected evolution of the community cat population in Spain under an ideal (but unrealistic) 80% sterilization scenario (2024–2049).

Populations	Year 0	Year 1	Year 2	Year 3	Year 4	Year 5	Year 6	Year 7	Year 8	Year 9	Year 10	Year 11	Year 12	Year 13	Year 14	Year 15	Year 16	Year 17	Year 18	Year 19	Year 20	Year 21	Year 22	Year 23	Year 24	Year 25
RL unneutered	560045	351239	221596	132241	84400	54532	34488	21483	14070	9202	6109	3781	2326	1554	995	630	400	264	156	88	57	38	24	15	9	6
RL neutered	140011	426064	561329	599642	584576	543591	492445	440811	388839	169549	106867	67768	44231	28277	17974	11615	7567	4860	3122	1927	1222	787	486	302	191	118
RL metapopulation	700056	777303	782926	731883	668975	598123	526933	462294	402908	178751	112976	71549	46557	29832	18969	12245	7967	5124	3278	2015	1279	824	510	318	201	123
RM unneutered	236724	164333	111136	79588	55329	37199	25616	17756	12423	8780	5952	3934	2660	1793	1205	867	578	404	270	193	137	87	72	51	38	25
RM neutered	59181	181732	246520	270810	272918	262988	244562	224835	203007	107617	74791	52155	35695	24644	17090	11732	8014	5397	3586	2481	1690	1158	818	572	393	281
RM metapopulation	295905	346064	357655	350398	328248	300187	270178	242591	215430	116397	80743	56090	38355	26436	18295	12600	8592	5801	3856	2674	1827	1246	889	622	431	305
RH unneutered	122402	91399	64237	47735	36402	25617	18299	14408	11063	8412	6485	4785	3593	2741	2085	1548	1058	837	608	457	391	287	217	159	120	97
RH neutered	30600	93572	130427	145342	147342	145597	136714	126625	116458	69434	51462	39011	29277	21939	16847	12883	9730	7311	5543	4115	3120	2345	1753	1295	970	747
RH metapopulation	153002	184971	194664	193078	183744	171214	155013	141033	127521	77845	57946	43795	32870	24679	18933	14432	10788	8148	6150	4573	3510	2632	1970	1454	1090	844
RVH unneutered	143361	122549	94540	74852	59898	49203	39467	32108	26114	19875	16032	12845	10389	8430	7068	5759	4776	3804	3104	2613	2305	1847	1519	1320	974	783
RVH neutered	35840	108994	160300	181347	187156	184657	178971	170894	162090	113265	91314	75134	61770	50540	41305	33894	27524	22209	18082	14907	12224	10103	8472	6911	5646	4615
RVH metapopulation	179201	231543	254840	256199	247054	233860	218438	203002	188205	133140	107346	87978	72159	58971	48373	39653	32300	26013	21187	17520	14529	11950	9990	8231	6620	5398
UL unneutered	82941	49515	28104	16024	9244	5286	3200	1895	1128	673	391	216	122	70	39	22	13	7	4	2	1	1	0	0	0	0
UL neutered	20735	63186	81909	85761	82748	75917	67745	60184	52509	19515	11125	6481	3814	2240	1344	787	465	266	149	82	47	27	15	8	5	3
UL metapopulation	103676	112700	110013	101785	91992	81203	70945	62078	53636	20188	11515	6696	3936	2310	1383	809	478	274	152	84	49	28	16	9	5	3
UM unneutered	128518	80795	49908	32293	20395	12773	8063	4860	3137	1920	1187	736	447	298	186	112	67	39	26	16	10	5	3	1	1	0
UM neutered	32192	98100	128144	136286	133551	124418	113801	101799	89780	39281	24790	15734	9833	6136	3782	2340	1487	909	573	358	221	140	84	50	30	18
UM metapopulation	160710	178895	178052	168579	153947	137190	121864	106659	92917	41201	25977	16471	10280	6434	3968	2452	1554	947	598	374	231	146	87	51	30	18
UH unneutered	70975	47391	31383	22018	14983	10270	6905	4474	2892	1924	1346	907	614	383	247	172	115	79	52	35	22	15	9	7	4	2
UH neutered	17743	54119	72285	78880	79395	76372	71168	64624	57504	28662	19564	13445	8904	5888	3893	2552	1691	1158	778	516	340	219	148	101	67	45
UH metapopulation	88718	101510	103668	100898	94378	86642	78073	69098	60396	30585	20910	14352	9518	6271	4140	2724	1806	1238	830	551	362	234	157	108	71	47
UVH unneutered	106181	75417	53839	39224	29074	21628	15464	10562	7345	4839	3382	2395	1732	1214	844	568	385	280	202	139	101	73	53	39	27	17
UVH neutered	26545	80614	109548	119412	118724	114500	108548	100597	91075	52606	38436	28407	20440	14436	10019	6945	4809	3307	2390	1680	1177	810	566	397	285	207
UVH metapopulation	132726	156030	163387	158636	147797	136128	124012	111160	98420	57445	41817	30802	22171	15649	10863	7513	5195	3587	2592	1819	1278	882	619	435	312	224

[a] Abbreviations:
RL, RM, RH, RVH = rural municipalities with low, medium, high, or very high reproductive potential;
UL, UM, UH, UVH = urban municipalities with low, medium, high, or very high reproductive potential.
